# Supplementary material for: Immunodiagnostic plasma amino acid residue biomarkers detect cancer early and predict treatment response
Source: Nat Commun. 2025 Jul 14;16:6474. doi: 10.1038/s41467-025-61685-2 (PMC12260101; doi:10.1038/s41467-025-61685-2)
Supplement: Supplementary file 1 — Supplementary Information [file 41467_2025_61685_MOESM1_ESM.pdf]

SUPPLEMENTARY INFORMATION

Immunodiagnostic plasma amino acid residue biomarkers detect cancer early and predict treatment response

Cong Tang[1,2,3]*, Patrícia Corredeira[1], Sandra Casimiro[1], Qi Shi[4], Qiwei Han[4], Wesley Sukdao[2], Ana Cavaco[1], Cecília Melo-Alvim[5], Carolina Ochôa Matos[6,9], Catarina Abreu[5], Steven Walsh[2], Gonçalo Nogueira-Costa[5], Leonor Ribeiro[5], Rita Sousa[5], Ana Lorena Barradas[1], João Eurico Fonseca[6,9], Luís Costa[1,5*], Emma V. Yates[2]*, Gonçalo J. L. Bernardes[1,7,8]*

1 GIMM - Gulbenkian Institute for Molecular Medicine; Avenida Prof. Egas Moniz, 1649-028, Lisboa, Portugal

2 Proteotype Diagnostics Ltd, Babraham Research Campus, Cambridge, CB22 3AT, UK

3 Xi'an Fengcheng Hospital, No.9 Fengcheng Third Road, Xi'an, Shaanxi, China

4 Nova School of Business and Economics, R. da Holanda 1, 2775-405 Carcavelos, Portugal

5 Serviço de Oncologia Médica, ULSSM, Unidade Local de Saúde de Santa Maria; Lisboa 1649-035, Portugal

6 Serviço de Reumatologia, ULS de Santa Maria, Centro Académico de Medicina de Lisboa, Lisbon, Portugal

7 Yusuf Hamied Department of Chemistry, University of Cambridge; Lensfield Road, Cambridge CB2 1EW, UK

8 Translational Chemical Biology Group, Spanish National Cancer Research Centre (CNIO), Madrid 28029, Spain

9 Faculdade de Medicina, Universidade de Lisboa, Centro Académico de Medicina de Lisboa, Lisbon, Portugal

*Corresponding author: G.J.L.B. (gb453@cam.ac.uk), E.V.Y. (emma@proteotype.com), L.C. (luis.costa@ulssm.min-saude.pt) and C.T. (cong.tang@gimm.pt)

Table of contents:

Supplementary Figures 1 to 13

Supplementary Tables 1 to 5

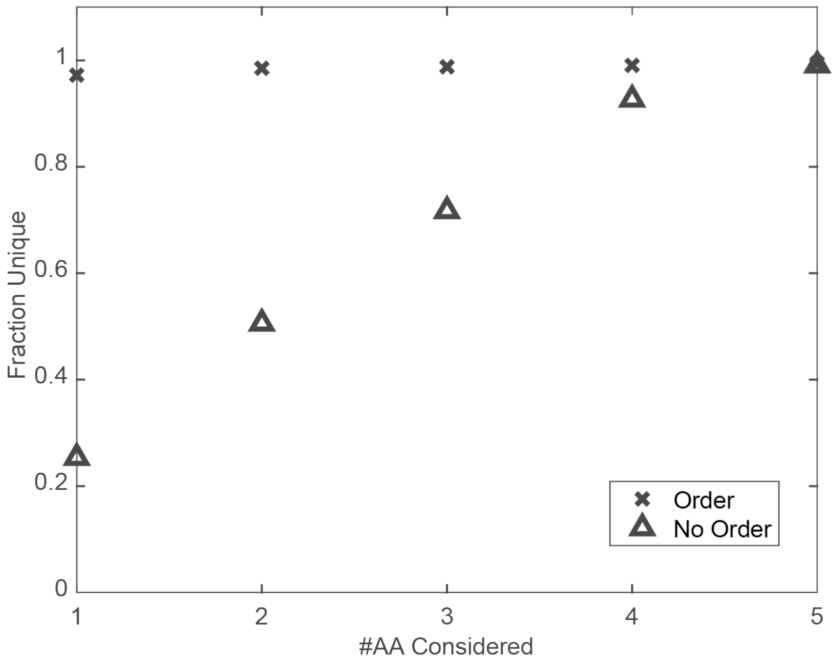

Supplementary Fig. 1 | Number of biomarkers required for identification using an Amino Acid Concentration Signature (AACS). We performed a bioinformatics analysis, examining the protein sequences of all 1700 proteins within the human plasma proteome, and determined the impact of the number of targeted amino acids on the uniqueness of protein sequences if the order of the amino acids was, or was not, included in the identification. We determined that if the order was included – such as in the protein fluorosequencing work of Swathimanian et al[1] – then only 2 amino acid types were required for uniqueness. If order was not included, as in AACS, then 5 amino acid types were required for uniqueness.

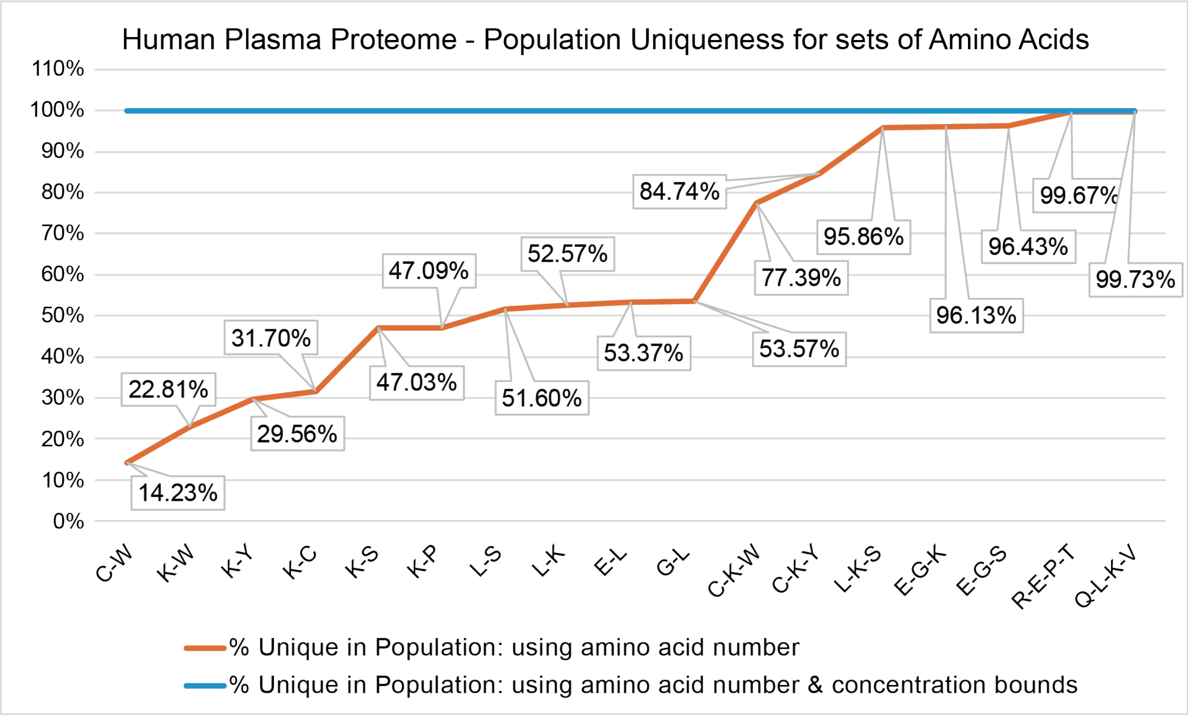

Supplementary Fig. 2 | Determination of the optimal AACS biomarkers for uniqueness. Given that there are 20 canonical amino acid residue types and 5 distinct amino acid residue types are required for uniqueness, there are 15,504 possible biomarker combinations (20 choose 5). To determine the optimal combination among synthetically accessible residues, we considered possible combinations of amino acids and how these combinations contributed to uniqueness of protein sequences, given that we were aiming to capture maximum information from the combined signal across all amino acid sequences. The results are shown below, for the case "without concentration bounds", where simply the protein sequences rather than the expected healthy bounds of their concentration within plasma is considered. We then considered for the high uniqueness couples – including and to the right of "C, K, W" – whether we could devise a strategy to uniquely target these amino acid types chemically. For example, the highest differentiation combination "Q, L, K, V" would require chemically and quantitatively labelling Leucine and Valine which is not possible via current chemistry. We determined that "C, K, W, and Y" would be possible for us to quantitatively chemically label due to the specific reactive chemical nature of those R-groups, while also offering excellent uniqueness among protein residues and differentiation between disease states.

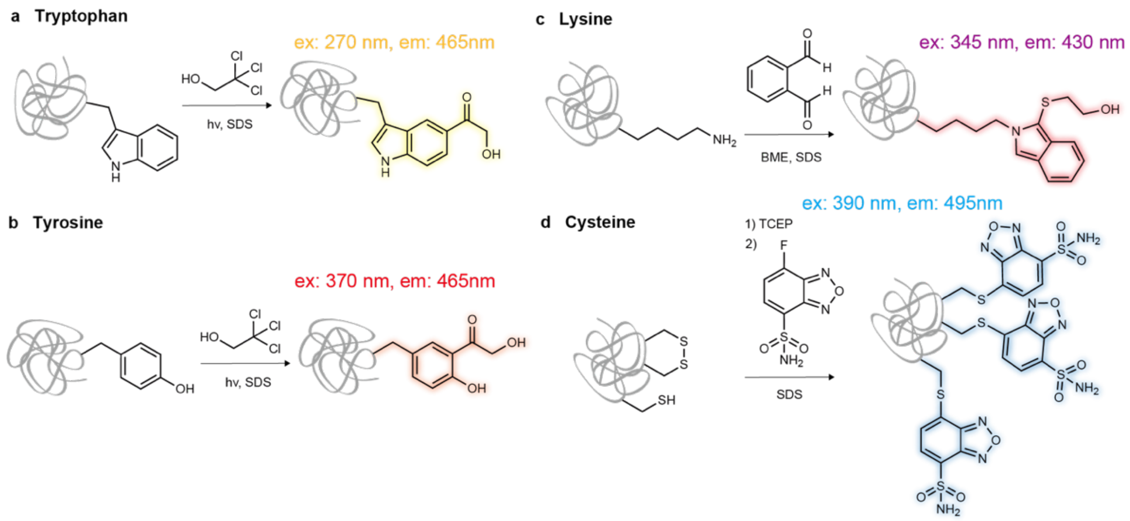

Supplementary Fig. 3 | Bioorthogonal labelling strategy reaction mechanisms. Each amino acid residue R-group, when fluorogenically labelled, generates a fluorescent species (coloured) which has a distinct pair of excitation (ex) and emission (em) wavelength. **a,** Photocatalysed, radical reaction with trichloroethanol installs a hydroxyethanone to the tryptophan ring, extending the conjugation and producing fluorescence in the visible region (465 nm emission). **b,** Photocatalysed radical reaction with trichloroethanol installs a hydroxyethanone to the tyrosine ring, extending the conjugation and producing fluorescence in the visible region (465 nm emission). In **a)** and **b)**, the hydroxyethanone can be appended to various positions on the phenyl / phenol ring. **c,** Reaction of a lysine primary amine group with phthalaldehyde involves attack on the first aldehyde, loss of water to form an imine, 2-mercaptoethanol then reveals the secondary amine for attack on the pendant aldehyde, and a second loss of water makes the reaction irreversible and extends the conjugation for fluorescence in the visible region. **d,** Where the cysteine is in a natively reduced state, labelling involves an addition elimination reaction where the cysteine thiol removes a quenching fluorine from the 7 sulfobenzofurazan, generating fluorescence. Where the cysteine is natively disulphide bonded, reduction with TCEP liberates two cysteine thiols for addition-elimination reactions, as shown in **d)**, and each cysteine is labelled with fluorescent 7-sulfobenzofurazan.

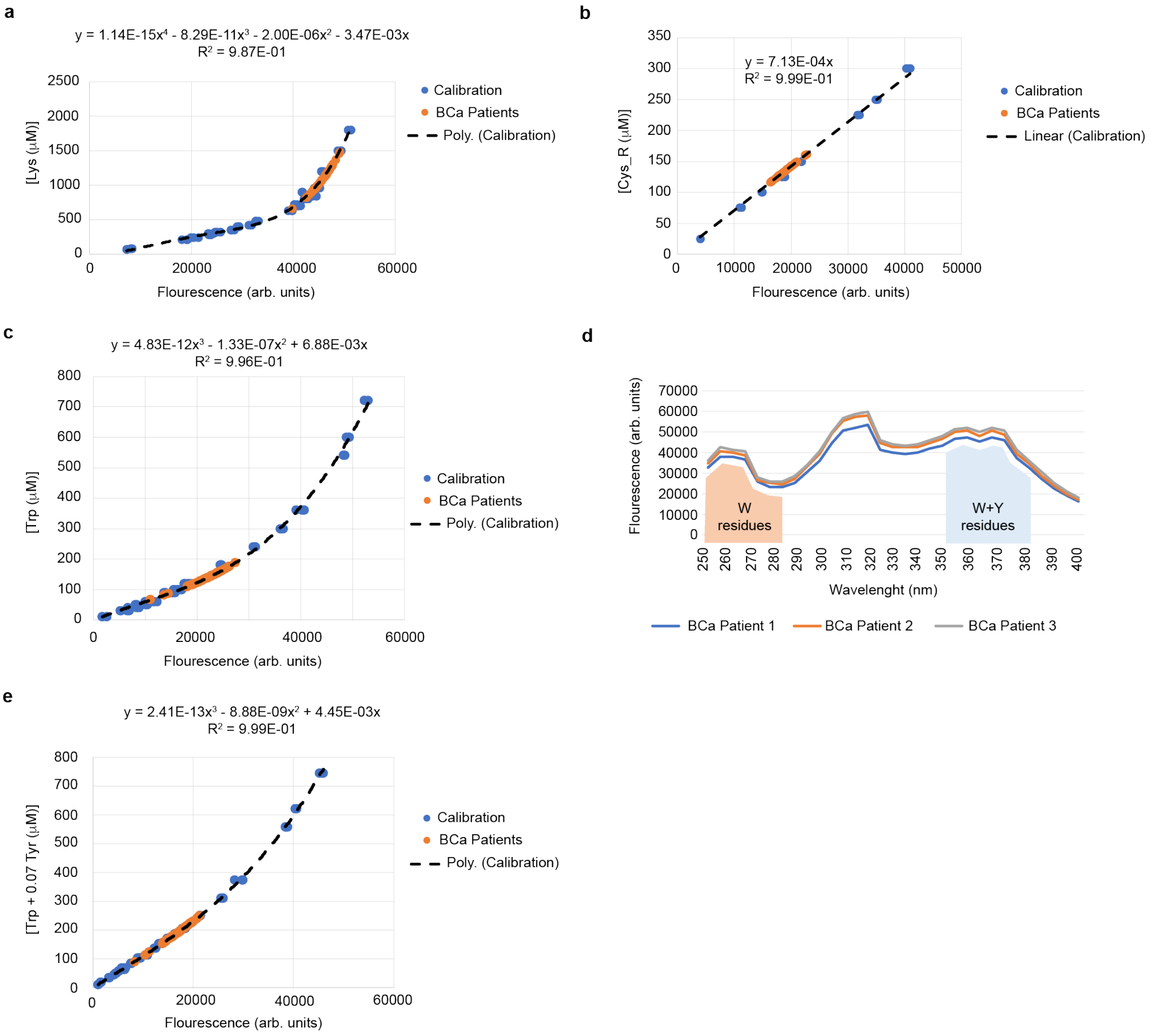

Supplementary Fig. 4 | Calibration curves used to transform the fluorescence intensity measured for patient samples into corresponding amino acid concentration. a, Calibration curve for Lysine (Lys), which determines the relationship between fluorescence intensity and the concentration of the Lys amino acid type, based on labelling Lys amino acid residues within a variety of protein standards. Lys amino acid residues within proteins are quantitatively labelled, meaning that they contribute to the single quantitative relationship between fluorescence intensity and Lys amino acid concentration. The calibration curve is then used to transform the measured fluorescence intensity for Breast Cancer (BCa) patient samples into the micromolar concentration of the Lys amino acid type in these patient samples. Shown for fluorescence measured at λ_{ex}=345 nm, λ_{em}=430 nm. **b,** Calibration curve for the Cys_R amino acid type measured at λ_{ex}=395 nm, λ_{em}=495 nm. **c,** Calibration curve for the Trp amino acid type, measured at λ_{ex}=270 nm, λ_{em}=465 nm. The Trp and Tyr labelling reactions are performed in one pot, and the Trp and Tyr amino acid types each contribute to distinct peaks in the resulting fluorescence spectra. The peak at λ_{ex}=270 nm, λ_{em}=465 nm is contributed by exclusively the Trp amino acid type, whereas the peak at λ_{ex}=370 nm, λ_{em}=465 nm has contributions from both the Trp and Tyr amino acid types. An optimization exercise was performed to identify that the

peak at λ_{ex}=370 nm, λ_{em}=465 nm describes [Trp + 0.07Tyr], where Trp is the concentration of the Trp amino acid type calculated using curve B, and Tyr is the concentration of the Tyr amino acid type to be solved for, both in micromolar. **d,** Calibration curve for the Tyr amino acid type, measured at λ_{ex}=370 nm, λ_{em}=465 nm. This curve describes [Trp + 0.07Tyr]. To solve for Tyr, the concentration of the Trp amino acid type determined via curve B is subtracted, and the resulting calculated 0.07Tyr amino acid concentration is divided by 0.07 to determine the concentration of the Tyr amino acid type in the solution.

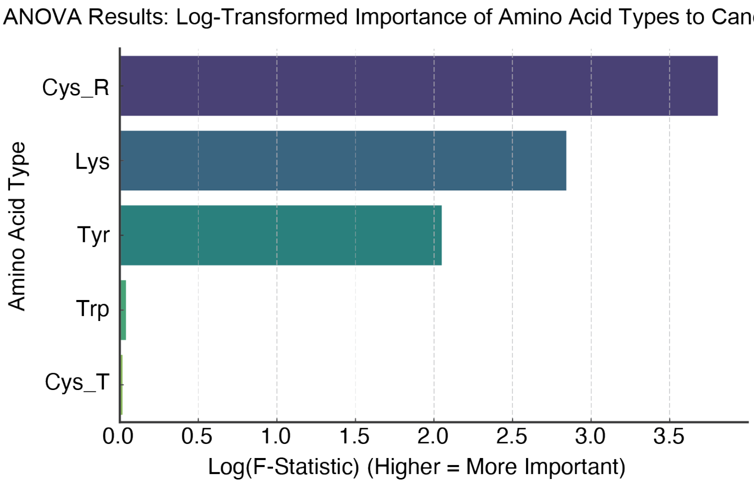

Amino Acid	F-Statistic	p-Value
Cys_R	4.4E+03	2E-09
Lys	1.62E+01	1.17E-04
Tyr	6.76E+00	1.08E-02
Trp	3.98E-02	8.42E-01
Cys_T	1.75E-02	8.95E-01

Supplementary Fig. 5 | Log-transformed F-statistics from ANOVA analysis of amino acid levels in cancer and healthy samples. Higher log(F-statistic) values indicate greater differentiation between cancer and healthy groups, suggesting a stronger association with cancer status. The amino acid Cys_R shows the highest significance, followed by Lys and Tyr, while Trp and Cys_T exhibit minimal differentiation. We clarify that while ANOVA is conventionally used with linear models, in our analysis ANOVA was applied not as a model inference tool, but to statistically assess the differentiation of individual amino acid levels between cancer and healthy groups.

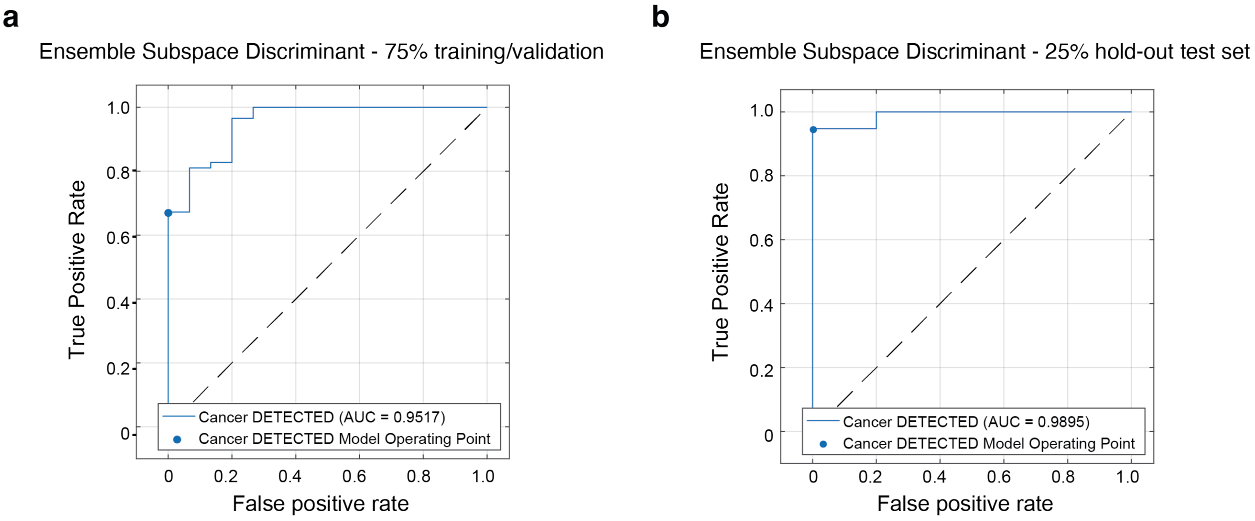

Supplementary Fig. 6 | Cancer detection using a held-out test set. a) To evaluate model performance using a held-out test set, we first randomized the dataset and assigned 75% (N=73) to a training and validation set, with the performance on the validation set estimated using 5X cross validation, resulting in the ROC curve shown (AUROC = 0.95). **b)** We assigned the remaining 24 data points to a held-out test set and used this to test model performance, resulting in the ROC curve shown (AUROC = 0.99).

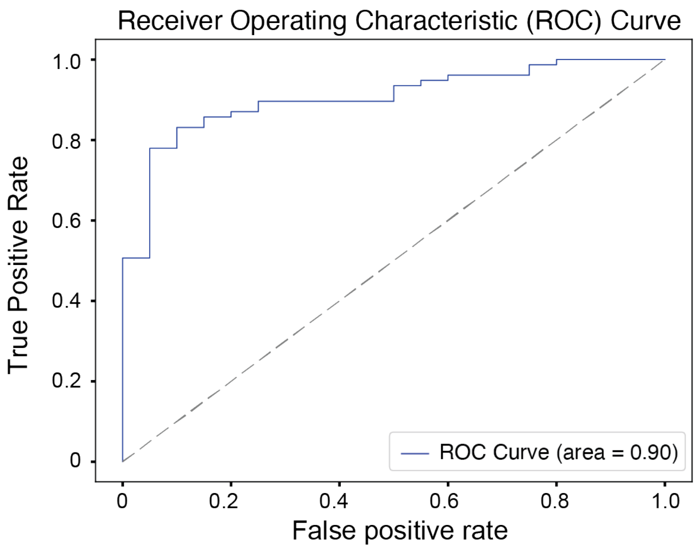

Supplementary Fig. 7 | Cancer detection using Leave-Out-One validation and a highly interpretable model. Following performing 5X Cross Validation to estimate cancer detection performance as in Figure 4b, we randomized the dataset and divided into N=97 folds for N=97 patient samples (biological replicates). We then trained a Support Vector Machine (SVM) classifier with a linear kernel and randomness disabled on N=96 data points and tested the performance on the remaining data point. We iterated this approach over all N=97 data points and averaged the AUROC to provide a combined performance metric.

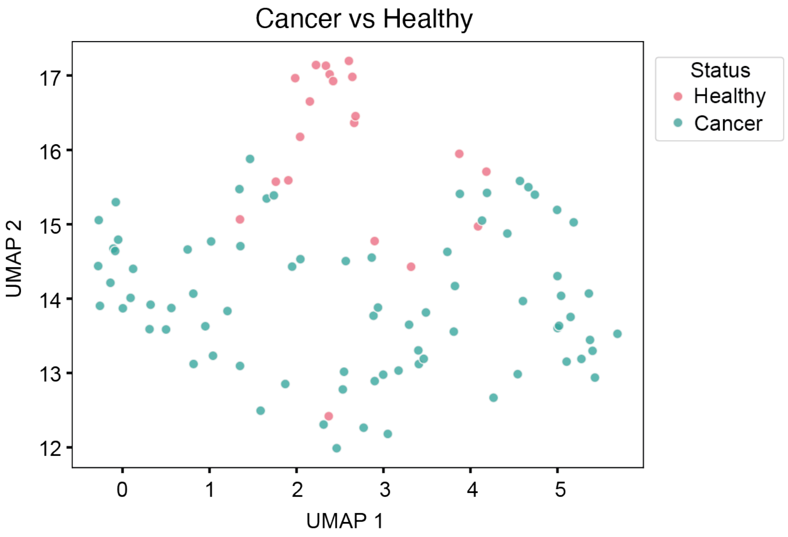

Supplementary Fig. 8 | UMAP visualization of AACS differentiating cancer and healthy samples. Each point represents an individual sample, with clustering patterns indicating similarities in amino acid distributions. Distinct group separations suggest meaningful biochemical differences between cancer and healthy states, supporting the potential utility of AACS profiling for classification.

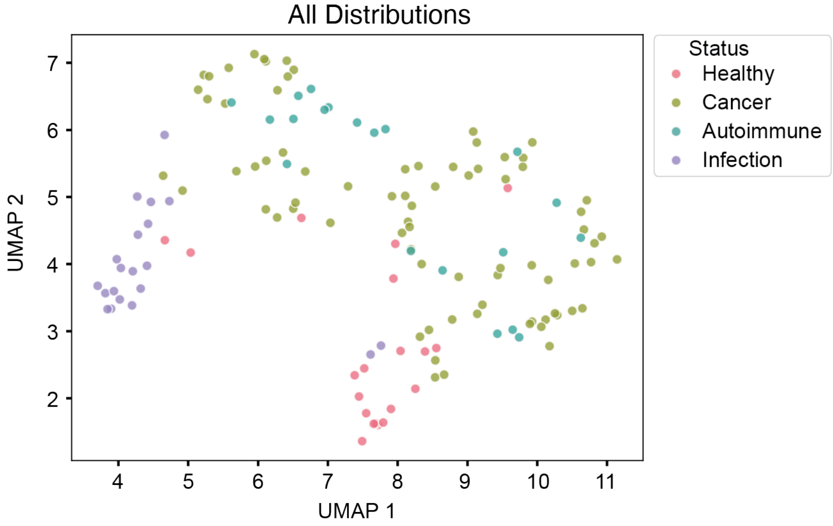

Supplementary Fig. 9 | UMAP representation of AACS profiles across different health conditions, including Healthy, Cancer, Autoimmune, and Infection groups. Each point represents an individual sample, with color-coded categories indicating distinct biological states. Clustering patterns suggest varying degrees of biochemical similarity, with distinct separations between Cancer, Healthy, and Infection groups. The partial overlap between Cancer and Autoimmune states, although differentiable, suggests that the immune system perceive these conditions as similar and further supports the host immune response as a major driver of AACS signals.

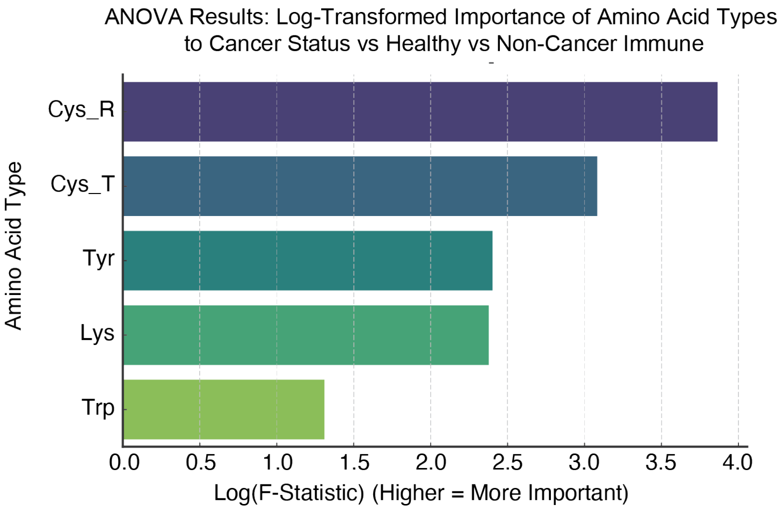

Amino Acid	F-Statistic	p-Value
Cys_R	4.67E+01	1.14E-20
Cys_T	2.08E+01	4.06E-11
Tyr	1E+01	5.2E-06
Lys	9.78E+00	7.12E-06
Trp	2.7E+00	4.8E-02

Supplementary Fig. 10 | Log-transformed F-statistics from ANOVA analysis comparing amino acid levels among cancer, healthy, and non-cancer immune samples. Higher log(F-statistic) values indicate greater differentiation between the groups, signifying a stronger association with group classification. The amino acid Cys_R shows the most significant variation, followed by Cys_T, Tyr, and Lys, while Trp exhibits the least differentiation. However, all amino acid types are statistically significant. As in **Extended Data Fig. 5**, ANOVA is used only to assess relative contribution of amino acid types, not as a model inference tool.

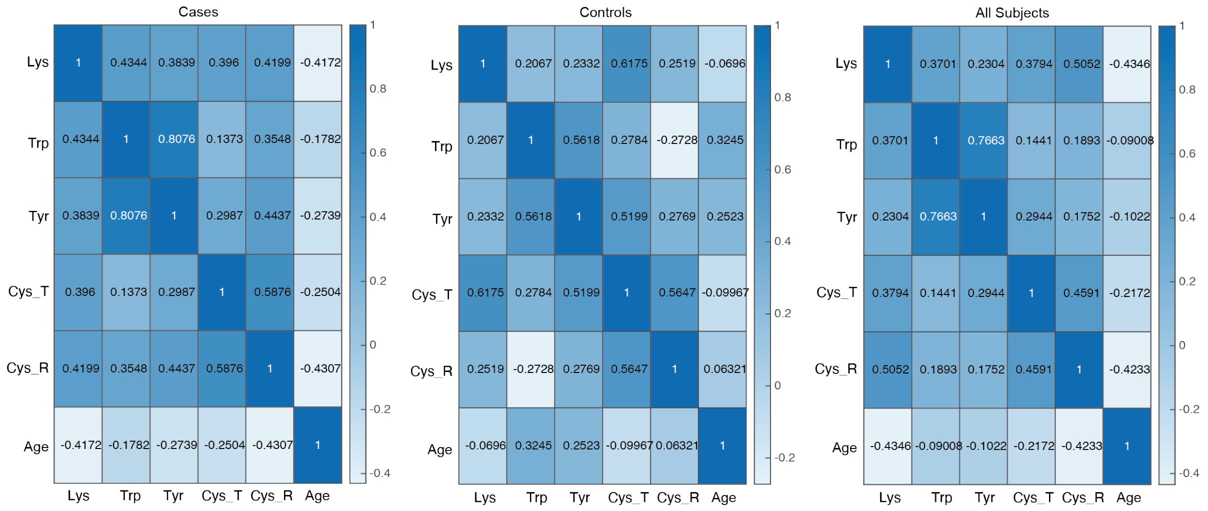

Supplementary Fig. 11 | Correlation matrices between Amino Acid residue levels and age as continuous variables for cases, controls, and the entire cohort. Each cell represents the Pearson correlation coefficient (r) between pairs of variables (Lys, Trp, Tyr, Cys_T, Cys_R amino acid levels and Age). The Pearson's r values are shown both colorimetrically and numerically. Possible values for Pearson's r range from -1 (perfect negative correlation), through 0 (no correlation) to 1 (perfect positive correlation). The left panel shows data from cancer cases, the middle panel shows controls, and the right panel shows all subjects combined. While some weak correlations with Age are observed (e.g., Lys and Cys_R negatively correlated in cases; Trp and Tyr weakly positively correlated in controls), none reach a level suggesting strong age-dependence.

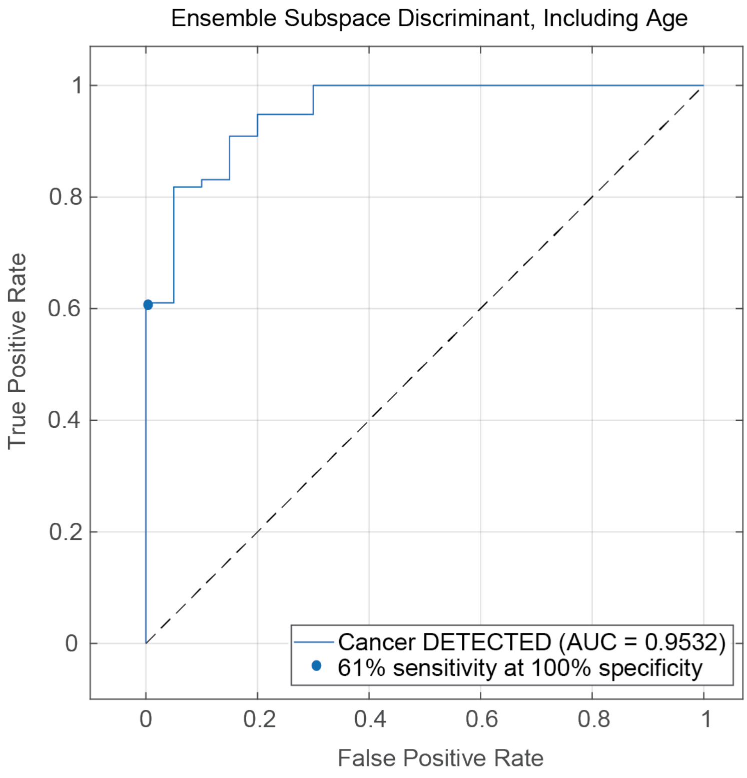

Supplementary Fig. 12 | ROC curve showing classifier performance when Age is included as a covariable. The same Ensemble Subspace Discriminant model used in the main analysis (Figure 4b) was retrained to include Age as a feature. The classifier achieves a comparable AUROC of 0.9532, but the **sensitivity at 100% specificity drops to 61%**, suggesting that inclusion of Age does not improve cancer detection performance and may introduce minor overfitting.

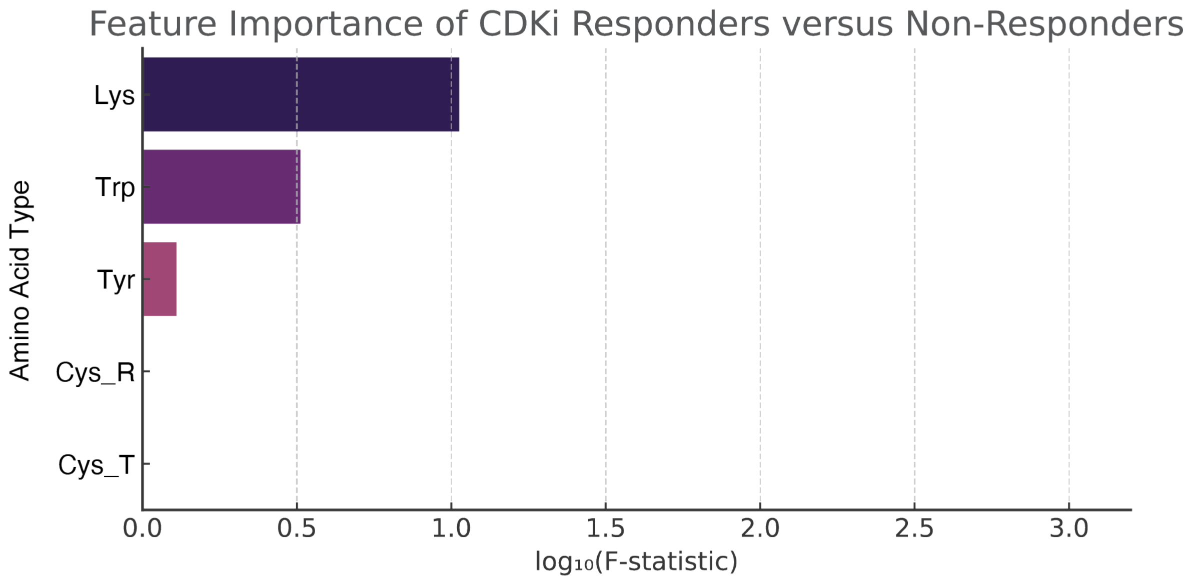

Supplementary Fig. 13 | Feature importance analysis comparing CDKi responders and non-responders. Log-transformed ANOVA F-statistics ($\log_{10}$ scale) are shown for each amino acid residue feature, reflecting their relative ability to differentiate between response groups. The features Lys, Trp, and Tyr contribute to the class separation, while other features show minimal contribution.

Supplementary Table 1. Fluorescence wavelengths of each labelling reaction.

	Emission scan		Excitation scan	
**Amino Acid**	**λ_{ex}, max (nm)**	**λ_{em}, max (nm)**	**λ_{ex}, max (nm)**	**λ_{em}, max (nm)**
Cysteine (Cys_T), Free Cysteine (Cys_R)	390	460-600	340-440	495
Lysine (Lys)	345	380-600	300-400	430
Tryptophan (Trp), Tyrosine (Tyr)	260; 370	340-600; 410-600	250-400	465

Supplementary Table 2: 96-well plate layout of standard protein samples for calibration plates measurement.

	1	2	3	4	5	6	7	8	9	10	11	12
A	60 µM BSA			60 µM Beta-Lac			120 µM LYZ			1 µM PH*		
B	50 µM BSA			50 µM Beta-Lac			100 µM LYZ			PBS		
C	45 µM BSA			45 µM Beta-Lac			90 µM LYZ			60 µM BSA		
D	30 µM BSA			30 µM Beta-Lac			60 µM LYZ			60 µM Beta-Lac		
E	25 µM BSA			25 µM Beta-Lac			50 µM LYZ			120 µM LYZ		
F	20 µM BSA			20 µM Beta-Lac			40 µM LYZ					
G	15 µM BSA			15 µM Beta-Lac			30 µM LYZ					
H	5 µM BSA			5 µM Beta-Lac			10 µM LYZ					

*PH wells are only required for the tryptophan/tyrosine labeling (Trp/Tyr labeling solution) because this is a control to deconvolute the fluorescence of those amino acid types. These wells were empty for all other labeling solutions to conserve this expensive protein.

Supplementary Table 3: Preparation of each labelling plate for calibration curves.

Procedures	Wells	Lys plate[#]	Trp/Tyr plate	Cys_R plate	Cys_T plate
1	Grey	100 µL of indicated protein solution			50 µL of indicated protein solution
2	Yellow	100 µL PBS			50 µL PBS
3	Green	100 µL of indicated protein solution			50 µL of indicated protein solution
4	Green	100 µL PBS			50 µL Cys_R solution
5	Yellow	100 µL Lys labeling solution	100 µL Trp/Tyr labeling solution	100 µL Cys_T labeling solution	50 µL Cys_R solution
6	Grey	100 µL Lys labeling solution	100 µL Trp/Tyr labeling solution	100 µL Cys_T labeling solution	50 µL Cys_R solution
7	Entire plate	NA	302 nm UV wavelength, at 100% intensity, irradiate for 30 mins[§]	Shaking for 45 min, plate covered and protected from light.	Wait 30 min, plate covered and protected from light.
8	Green	NA			100 µL PBS
9	Yellow	NA			100 µL Cys_T labeling solution
10	Grey	NA			100 µL Cys_T labeling solution
11	Entire plate	NA			Wait 45 min, plate covered and protected from light.
Measurement	Single wavelength	Excitation at 345 nm, Emission at 430 nm.	NA		
	Emission scan	Excite at 345 nm, measure	Excite at 260 nm, measure	Excite at 390 nm, measure	Excite at 390 nm, measure

		emission from 380-600 nm.	emission from 340-600 nm.<br>Excite at 370 nm, measure emission from 410-600 nm.	emission 460-600 nm.	emission 460-600 nm.
	Excitation scan	Excite from 300-400 nm, measure emission at 430 nm.	Excite from 250-400 nm, measure emission at 465 nm.	Excite from 340-440 nm, measure emission at 495 nm.	Excite from 340-440 nm, measure emission at 495 nm.
Step size		10 nm	5 nm		
Manual gain setting[*] (Record from plate reader)		A1-A3 (Tecan M200 - 83)	A7-A9 (Tecan M200 - 100)	A1-A6 (Tecan M200 - 100; Tecan Spark - 81)	A1-A3 (Tecan M200 - 97; Tecan Spark - 66)

[#]The products we are measuring in the Lys labeling reaction degrade over time, so the samples are measured as quickly as possible after the Lys labeling solution is added to the wells.

[§] The irradiation was performed by a UV transilluminator (UVITEC Cambridge, SXT-F20.M, 6x15 W), at 302 nm UV wavelength setting and 100% (maximum) intensity.

[*]A manual gain setting was chosen such that the highest specific protein concentration on the plate, from indicated wells, does not saturate. The manual gain setting was recorded and used for all other labeling reaction measurements, including for the clinical samples. Bottom reading was chosen for all the fluorescence measurement.

Supplementary Table 4: 96-well plate layout of patient samples for every labelling reaction measurement.

	1	2	3	4	5	6	7	8	9	10	11	12
A	Pt.#1[&]			Pt.#9			Pt.#17			Dye		
B	Pt.#2			Pt.#10			Pt.#18			Pt.#1	Pt.#2	Pt.#3
C	Pt.#3			Pt.#11			Pt.#19			Pt.#4	Pt.#5	Pt.#6
D	Pt.#4			Pt.#12			Pt.#20			Pt.#7	Pt.#8	Pt.#9
E	Pt.#5			Pt.#13			30 μM BSA			Pt.#10	Pt.#11	Pt.#12
F	Pt.#6			Pt.#14			30 μM Beta-Lac			Pt.#13	Pt.#14	Pt.#15
G	Pt.#7			Pt.#15			60 μM LYZ			Pt.#16	Pt.#17	Pt.#18
H	Pt.#8			Pt.#16			10 μM PH[*]			Pt.#19	Pt.#20	PBS

[*]PH wells are only required for the tryptophan/tyrosine labeling (Trp/Tyr labeling solution) because this is a control to deconvolute the fluorescence of those amino acid types. These wells were empty for all other labeling solutions to conserve this expensive protein.
[&]Pt. means Patient.

Supplementary Table 5: Preparation of each labelling plate for patient samples.

Procedures	Wells	Lys plate[#]	Trp/Tyr plate	Cys_R plate	Cys_T plate
1	Blue	100 µL of indicated patient sample			50 µL of indicated patient sample
2	Grey	100 µL of indicated standard protein solution			50 µL standard protein solution
3	Yellow	100 µL PBS			50 µL PBS
4	Green	100 µL of indicated patient sample			50 µL of indicated patient sample
5	Green	100 µL PBS			50 µL Cys_R solution
6	Yellow	100 µL Lys labeling solution	100 µL Trp/Tyr labeling solution	100 µL Cys_T labeling solution	50 µL Cys_R solution
7	Grey	100 µL Lys labeling solution	100 µL Trp/Tyr labeling solution	100 µL Cys_T labeling solution	50 µL Cys_R solution
8	Blue	100 µL Lys labeling solution	100 µL Trp/Tyr labeling solution	100 µL Cys_T labeling solution	50 µL Cys_R solution
9	Entire plate	NA	302 nm UV wavelength, at 100% intensity, irradiate for 30 mins	Shaking for 45 min, plate covered and protected from light.	Wait 30 min, plate covered and protected from light.
10	Green	NA			100 µL PBS
11	Yellow	NA			100 µL Cys_T labeling solution
12	Grey	NA			100 µL Cys_T labeling solution
13	Blue	NA			100 µL Cys_T labeling solution

<table>
<tr><td>14</td><td>Entire plate</td><td colspan="3">NA</td><td>Wait 45 min, plate covered and protected from light.</td></tr>
<tr><td rowspan="4">Measurement</td><td>Single wavelength</td><td>Excitation at 345 nm, Emission at 430 nm.</td><td colspan="3">NA</td></tr>
<tr><td rowspan="2">Emission scan</td><td rowspan="2">Excite at 345 nm, measure emission from 380-600 nm.</td><td>Excite at 260 nm, measure emission from 340-600 nm.</td><td rowspan="2">Excite at 390 nm, measure emission 460-600 nm.</td><td rowspan="2">Excite at 390 nm, measure emission 460-600 nm.</td></tr>
<tr><td>Excite at 370 nm, measure emission from 410-600 nm.</td></tr>
<tr><td>Excitation scan</td><td>Excite from 300-400 nm, measure emission at 430 nm.</td><td>Excite from 250-400 nm, measure emission at 465 nm.</td><td>Excite from 340-440 nm, measure emission at 495 nm.</td><td>Excite from 340-440 nm, measure emission at 495 nm.</td></tr>
<tr><td>Step size</td><td></td><td>10 nm</td><td colspan="3">5 nm</td></tr>
<tr><td>Manual gain setting*</td><td></td><td>Tecan M200 - 83</td><td>Tecan M200 - 100</td><td>Tecan M200 - 100; Tecan Spark - 81</td><td>Tecan M200 - 97; Tecan Spark - 66</td></tr>
</table>

*The manual gain setting was identified using the calibration plate for each labelling reaction measurement.

REFERENCES

1. Swaminathan, J. et al. Highly parallel single-molecule identification of proteins in zeptomole-scale mixtures. *Nat. Biotechnol.* **36**, 1076–1082 (2018).
