# Supplementary material for: Immunodiagnostic plasma amino acid residue biomarkers detect cancer early and predict treatment response
Source: Nat Commun. 2025 Jul 14;16:6474. doi: 10.1038/s41467-025-61685-2 (PMC12260101; doi:10.1038/s41467-025-61685-2)
Supplement: Supplementary file 2 — Description of Addtional Supplementary File [file 41467_2025_61685_MOESM2_ESM.pdf]

Description of Additional Supplementary File

Supplementary Data 1 - Excel file containing patient sample information. Clinical and demographic information for all patient participants included in the study. This Excel file contains details such as age, sex, diagnosis, treatment history, and sample classification for everyone, as used in the analyses described in the manuscript.
