# Supplementary material for: Immunodiagnostic plasma amino acid residue biomarkers detect cancer early and predict treatment response
Source: Nat Commun. 2025 Jul 14;16:6474. doi: 10.1038/s41467-025-61685-2 (PMC12260101; doi:10.1038/s41467-025-61685-2)
Supplement: Supplementary file 4 — Reporting Summary [file 41467_2025_61685_MOESM4_ESM.pdf]

nature portfolio

Corresponding author(s): Gonçalo Bernardes, Emma Yates, Luis Costa, Cong Tang

Last updated by author(s): Jun 8, 2025

Reporting Summary

Nature Portfolio wishes to improve the reproducibility of the work that we publish. This form provides structure for consistency and transparency in reporting. For further information on Nature Portfolio policies, see our Editorial Policies and the Editorial Policy Checklist.

Statistics

For all statistical analyses, confirm that the following items are present in the figure legend, table legend, main text, or Methods section.

n/a	Confirmed	
☐	☒	The exact sample size (*n*) for each experimental group/condition, given as a discrete number and unit of measurement
☐	☒	A statement on whether measurements were taken from distinct samples or whether the same sample was measured repeatedly
☐	☒	The statistical test(s) used AND whether they are one- or two-sided<br>*Only common tests should be described solely by name; describe more complex techniques in the Methods section.*
☐	☒	A description of all covariates tested
☐	☒	A description of any assumptions or corrections, such as tests of normality and adjustment for multiple comparisons
☐	☒	A full description of the statistical parameters including central tendency (e.g. means) or other basic estimates (e.g. regression coefficient) AND variation (e.g. standard deviation) or associated estimates of uncertainty (e.g. confidence intervals)
☐	☒	For null hypothesis testing, the test statistic (e.g. *F*, *t*, *r*) with confidence intervals, effect sizes, degrees of freedom and *P* value noted<br>*Give P values as exact values whenever suitable.*
☒	☐	For Bayesian analysis, information on the choice of priors and Markov chain Monte Carlo settings
☐	☒	For hierarchical and complex designs, identification of the appropriate level for tests and full reporting of outcomes
☐	☒	Estimates of effect sizes (e.g. Cohen's *d*, Pearson's *r*), indicating how they were calculated

Our web collection on statistics for biologists contains articles on many of the points above.

Software and code

Policy information about availability of computer code

Data collection	Fluorescence signals reading were performed on Tecan Infinite 200 Pro microplate reader and Tecan Spark multimode microplate reader. All staststical analyses were performed using the MATLAB (R2023a) or Python programming language (version 3.10.9).
Data analysis	The linear discriminant, quadratic discriminant, ensemble subspace discriminant, and weighted K-nearest neighbor classifiers were performed and evaluated. MANOVA methods were conducted using the 'MANOVA' function from the statsmodels package.<br>All statistical analyses were performed using the MATLAB (R2023a) or Python programming language (version 3.10.9). MANOVA methods were conducted using the 'MANOVA' function from the statsmodels package. No statistical method was used to predetermine sample size. All collected data were included in the analyses, and no data were excluded. Randomization and blinding were not applied, as they were not applicable to the design and objectives of this study. Detailed sample sizes and statistical test results are provided in the figure legends and relevant sections of the manuscript.

For manuscripts utilizing custom algorithms or software that are central to the research but not yet described in published literature, software must be made available to editors and reviewers. We strongly encourage code deposition in a community repository (e.g. GitHub). See the Nature Portfolio guidelines for submitting code & software for further information.

Data

Policy information about availability of data

All manuscripts must include a data availability statement. This statement should provide the following information, where applicable:

- Accession codes, unique identifiers, or web links for publicly available datasets
- A description of any restrictions on data availability
- For clinical datasets or third party data, please ensure that the statement adheres to our policy

All data supporting the findings of this study are available within the article and its Supplementary Information. Source data underlying the graphs and charts in the main figures are provided as a Source Data file. No additional datasets require deposition in a public repository. The custom software used to generate the analyses and figures in this study is commercially proprietary. In accordance with the Nature Communications Data Availability policy, it is nonetheless available to qualified researchers for academic (non-commercial) purposes upon request. To obtain access, please email the corresponding author at gb453@cam.ac.uk.

Research involving human participants, their data, or biological material

Policy information about studies with human participants or human data. See also policy information about sex, gender (identity/presentation), and sexual orientation and race, ethnicity and racism.

Reporting on sex and gender	105 patients in the study were female, and 65 patients were male.<br>Sex and/or gender was not restricted during participant recruitment. Sex was self-reported at enrolment. Although sex/gender was not a factor in the study design or inclusion criteria, we conducted sex- and/or gender-based analyses where applicable. Disaggregated data by sex are provided in the Source Data file, and overall participant numbers are reported in the Methods and Reporting Summary.
Reporting on race, ethnicity, or other socially relevant groupings	Race was reported as white, black and others.
Population characteristics	N=20 volunteer healthy donors, N=20 colorectal cancer patients, N=20 prostate cancer patients, N=17 pancreatic cancer patients, N=53 breast cancer patients (33 of them are for CDK4/6i study), N=20 Infection disease patients (COVID-19), N=20 autoimmune disease patients (Rheumatoid Arthitis).<br>HR/HER2- breast cancer patients were included, treated with CDK4/6i in either first- or second-line in combination with an aromatase inhibitor (AI) or fulvestrant until disease progression or unacceptable toxicity.
Recruitment	Samples were requested from Biobanco-GIMM, Lisbon Academic Medical Center, Lisbon, Portugal. Biobanco collect and store samples under standardized protocols with prior informed consent for future research use. As such, participants were not prospectively recruited for this specific study.Given the use of pre-existing biobank samples, there may be inherent limitations such as selection bias toward individuals who consented to sample storage, or underrepresentation of certain demographic or clinical subgroups. However, all samples included in the study met consistent inclusion criteria, and data were analyzed across multiple cohorts to mitigate bias. We believe these factors reduce the likelihood of systematic bias significantly affecting our main conclusions.<br>Cancer patients followed at CHULN-Hospital Santa Maria, Lisbon at baseline (before starting new line of therapy) according to the Oncology Department guidelines for disease evaluation.<br>Peripheral blood samples were collected from all participants at the time of metastatic diagnosis and before starting treatment (baseline).
Ethics oversight	All samples were collected with informed consent and approved by the Ethical Board of the Academic Medical Center of Lisbon CAML(CHLN/FMUL/IMM). Experiments are conformed to the principles set out in the WMA Declaration of the Helsinki and the Department of Health and Human Services Belmont Report.<br>The study of HR/HER2- breast cancer patients with CDK4/6i treatment was carried out following the principles of the Declaration of Helsinki, good clinical practice guidelines, and local regulations. The Ethics Committee of Centro Hospitalar Universitário Lisboa Norte (CHULN) approved the study protocol (approval number 343), and all participants provided written informed consent.

Note that full information on the approval of the study protocol must also be provided in the manuscript.

Field-specific reporting

Please select the one below that is the best fit for your research. If you are not sure, read the appropriate sections before making your selection.

☒ Life sciences ☐ Behavioural & social sciences ☐ Ecological, evolutionary & environmental sciences

For a reference copy of the document with all sections, see nature.com/documents/nr-reporting-summary-flat.pdf

Life sciences study design

All studies must disclose on these points even when the disclosure is negative.

Sample size	A total of 170 individuals were involved in the study.

Sample size	N=20 volunteer healthy donors, N=20 colorectal cancer patients, N=20 prostate cancer patients, N=17 pancreatic cancer patients, N=53 breast cancer patients (33 of them are for CDKi study), N=20 Infection disease patients (COVID-19), N=20 autoimmune disease patients (Rheumatoid Arthitis).<br>As the study is exploratory in nature, no statistical method was used to predetermine sample size, and all collected data were included in the analyses.
Data exclusions	No data were excluded from the analyses.
Replication	All patient samples were measured in three replicates.
Randomization	Randomization and blinding were not applied, as they were not deemed applicable to the study design.
Blinding	All analysis were blind to the condition during the data collection. Unblinding was done when the data was analyzed.

Reporting for specific materials, systems and methods

We require information from authors about some types of materials, experimental systems and methods used in many studies. Here, indicate whether each material, system or method listed is relevant to your study. If you are not sure if a list item applies to your research, read the appropriate section before selecting a response.

Materials & experimental systems

n/a	Involved in the study
☒	☐ Antibodies
☐	☒ Eukaryotic cell lines
☒	☐ Palaeontology and archaeology
☐	☒ Animals and other organisms
☒	☐ Clinical data
☒	☐ Dual use research of concern
☒	☐ Plants

Methods

n/a	Involved in the study
☒	☐ ChIP-seq
☒	☐ Flow cytometry
☒	☐ MRI-based neuroimaging

Eukaryotic cell lines

Policy information about cell lines and Sex and Gender in Research

Cell line source(s)	Mouse colon adenocarcinoma MC38 cell was purchased from Kerafast (ENH204-FP). Mouse melanoma cell line B16F10 was a gift from Dr Karine Marie Serre and Dr Susana Constantino Santos from iMM.
Authentication	All cell lines were authenticated using STR profiling.
Mycoplasma contamination	All cell lines were routinely tested for mycoplasma contamination using MycoAlert Mycoplasma Detection kit (Lonza, LT07-710). No contamination was detected during the study period.
Commonly misidentified lines<br>(See ICLAC register)	None of the cell lines used are listed in the ICLAC database of misidentified cell lines.

Animals and other research organisms

Policy information about studies involving animals; ARRIVE guidelines recommended for reporting animal research, and Sex and Gender in Research

Laboratory animals	8-week-old female and male C57BL/6J mice (purchased from Charles River) were used in this study.
Wild animals	This study did not involve wild animals.
Reporting on sex	female and male
Field-collected samples	This study did not involve samples collected from the field.
Ethics oversight	Animal work was performed in strict accordance with Portuguese Law (Portaria 1005/92) and the European Guideline 86/609/EEC and follow the Federation of European Laboratory Animal Science Associations guidelines and recommendations concerning laboratory animal welfare. All animal experiments were approved by the Portuguese official veterinary department for welfare licensing – Direção Geral de Alimentação e Veterinária (DGAV) and the IMM Animal Ethics Committee (authorization AWB_2021_03_GB_Targ CancerDrugs).<br>Animals were observed regularly; tumours were measured as described before and mouse weight was evaluated throughout the study. Mice were monitored regularly, and animals were euthanized by isoflurane overdose once tumour volume reached 1,000 mm^3, in accordance with the institutional animal care guidelines. The maximal tumour size/burden permitted by the ethics committee was 1,000 mm^3, and this limit was not exceeded during the study. No signs of animal sufferring or discomfort were

obseved, including no weight loss. The light/dark cycle was 14 h light/10 h dark (lights on at 07:00; lights off at 21:00). The temperature was 20-24 °C and the relative humidity was 55 ± 10%, with controlled supply of High Efficiency-Particulate Air (HEPA) filtered air provided to individually ventilated cages. Maximum number of animals per cage was 5. Social isolation was avoided whenever possible. The type of food was autoclaved diet pellets RM3A (P), from SDS Special Diets Services (Product code: 801030). Food was placed in a grid inside the cage and provided ad libitum to animals. The type of water was sterile water treated by reverse osmosis. Water was provided ad libitum to animals through bottles with a capillary hole.

Note that full information on the approval of the study protocol must also be provided in the manuscript.

Plants

Seed stocks

Report on the source of all seed stocks or other plant material used. If applicable, state the seed stock centre and catalogue number. If plant specimens were collected from the field, describe the collection location, date and sampling procedures.

Novel plant genotypes

Describe the methods by which all novel plant genotypes were produced. This includes those generated by transgenic approaches, gene editing, chemical/radiation-based mutagenesis and hybridization. For transgenic lines, describe the transformation method, the number of independent lines analyzed and the generation upon which experiments were performed. For gene-edited lines, describe the editor used, the endogenous sequence targeted for editing, the targeting guide RNA sequence (if applicable) and how the editor was applied.

Authentication

Describe any authentication procedures for each seed stock used or novel genotype generated. Describe any experiments used to assess the effect of a mutation and, where applicable, how potential secondary effects (e.g. second site T-DNA insertions, mosiacism, off-target gene editing) were examined.
